# NINJ1: implications for plasma membrane rupture and disease

**DOI:** 10.1042/BST20260711

**Published:** 2026-09-18

**Authors:** Elliott M. Bernard, Ella Hartenian

**Affiliations:** Department of Immunobiology, University of Lausanne, Epalinges, Switzerland

**Keywords:** cell death, immunology, inflammation, Ninj1

## Abstract

Cell death pathways are ancient strategies that maintain homeostasis and protect organisms from infection and injury. Lytic cell death culminates in the rupture of the plasma membrane, which was recently revealed to be driven by Ninjurin-1 (NINJ1), a plasma membrane protein that oligomerizes during cell death. NINJ1 oligomerization creates lesions in the plasma membrane that facilitate the release of cytosolic contents. In the present review, we discuss what is currently known about NINJ1 biology, focusing on the most current research into potential mechanisms of activation. We then highlight how NINJ1 has been implicated in specific disease contexts and discuss potential ways NINJ1 may contribute to disease. We conclude with the remaining open questions relating to NINJ1 biology.

## Introduction

**Figure 1:**
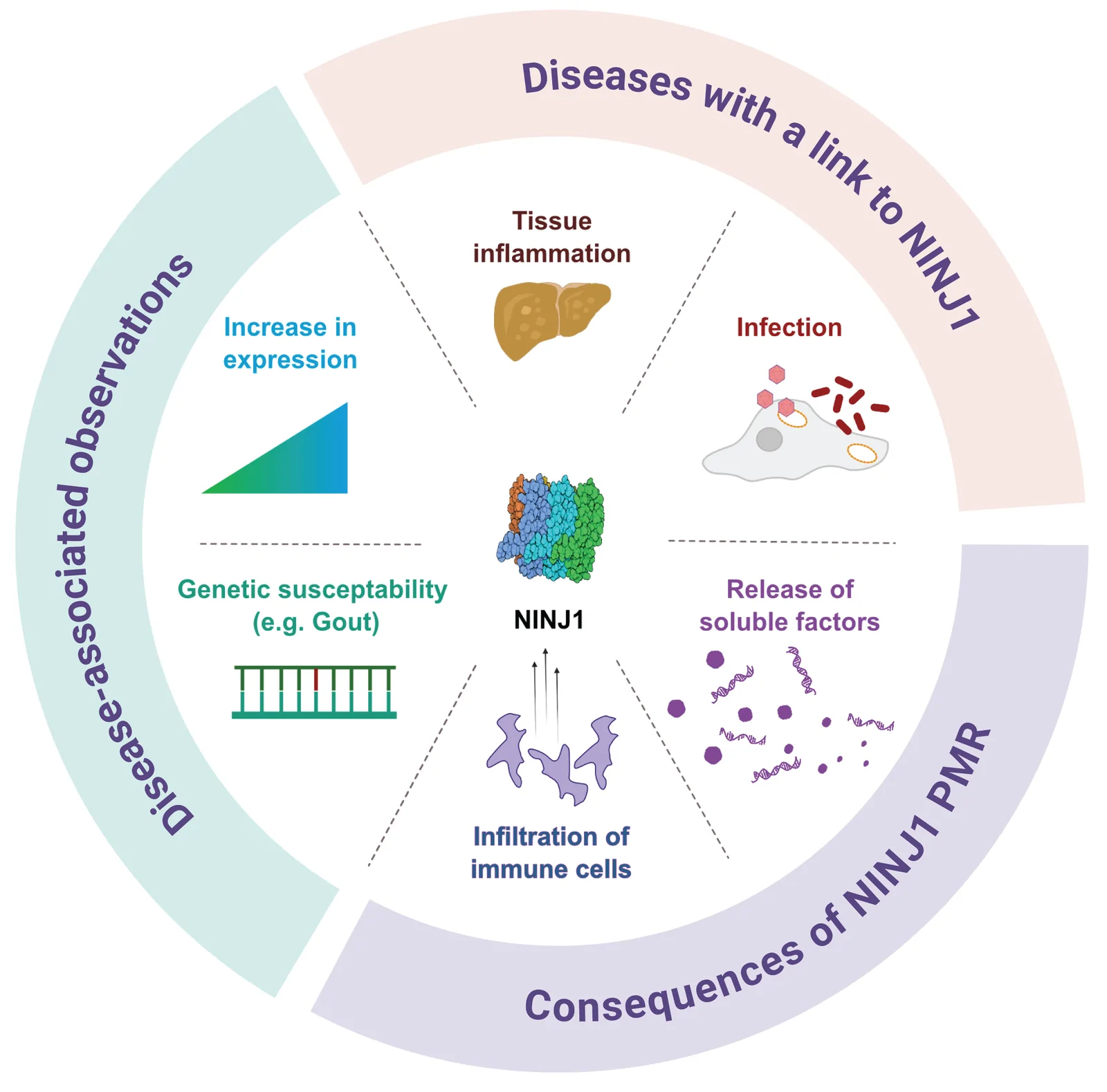
Functional consequences and disease associations of NINJ1 An overview of NINJ1 disease associations, highlighting the contexts in which NINJ1 has been implicated in disease, the consequences of this activation, and observations on the modulation of NINJ1 during disease.

Following insult or injury, including pathogen infection, cells can undergo several forms of regulated cell death to eliminate the infected or defective cell and initiate a protective immune response. Of these, lytic forms of regulated cell death include pyroptosis, necroptosis, ferroptosis, and secondary necrosis. All of these can cause inflammation by releasing pro-inflammatory molecules, including damage-associated molecular patterns (DAMPs), and recruiting immune cells, including neutrophils [1]. The release of small DAMPs and select inflammatory cytokines (ATP, IL-1b, and IL-18) can be achieved through small pores formed during cell death, for example by proteins in the gasdermin family that execute pyroptosis [2]. However, the release of larger DAMPs, like high mobility group box 1 protein (HMGB1), requires the rupture of the plasma membrane to produce larger lesions. For many years plasma membrane rupture (PMR) was thought to be a passive process driven by osmotic imbalance, exemplified by the characteristic cell swelling observed during lytic cell death. However, in 2021, Kayagaki et al. identified the plasma membrane protein Ninjurin-1, or NINJ1, as responsible for cell lysis, the terminal event in cell death [3]. Indeed, dying cells deficient for NINJ1 exhibit a persistent ballooned morphology and fail to release large cytoplasmic DAMPs. This has been observed for multiple forms of cell death including pyroptosis, secondary necrosis, ferroptosis, and necrosis from hydrogen peroxide [3–5]. Intriguingly, NINJ1-deficient cells do not show defects in lysis after necroptosis, highlighting a lytic form of death independent of NINJ1 [4]. Further work demonstrated that blocking NINJ1 activation with neutralizing antibodies improved outcomes in mice with viral or TNF-induced hepatitis [3,6]. These studies raised the exciting possibility that cell death-associated inflammation could be limited by targeting NINJ1. In the present review, we discuss the evidence for the proposed models for execution of PMR by NINJ1 and highlight the emerging role of NINJ1 in multiple disease contexts (Figure 1).

## NINJ1: from cell adhesion to cell death

NINJ1 is a highly conserved 16 kDa protein with three transmembrane alpha helices and unstructured N and C termini. Before its implication in cell death, NINJ1 was previously described as up-regulated in Schwann cells after axonal injury. Schwann cells are a key cell type that facilitates axon regeneration, and NINJ1 was identified through a cDNA library screen of mRNA up-regulated after axonal injury [7]. Mechanistically, the authors attributed this to a cell adhesion role for NINJ1 based on its plasma membrane localization and the induction of clustering of Jurkat cells upon NINJ1 overexpression [8]. Importantly, NINJ1 overexpression in HEK cells is now known to be cytotoxic [3,9], and a cell adhesion role has yet to be confirmed following the discovery of the novel, cell death-related function of NINJ1. That said, mutants that block NINJ1-induced cell clustering fall outside the structured region of the protein, and their effect on the cell death role of NINJ1 has not been investigated. In addition, the cell adhesion function of NINJ1 requires an extracellularly exposed N terminus, whereas the most recent structural insights suggest that the N terminus of NINJ1 is intracellular during homeostasis [7,8,10]. Further work is required to reconcile these discrepancies.

In living cells, NINJ1 forms an autoinhibited homodimer with all three helices in a straight conformation [10]. Upon cell death and activation, NINJ1 undergoes a profound structural change, oligomerizing to form a filament that is stabilized by the kinking of the first transmembrane helix (TM1), leading to intermolecular contacts between one NINJ1 molecule and its neighboring protomer [9,11,12]. While the structural transition from autoinhibited NINJ1 to active, oligomeric NINJ1 is well understood, the mechanism behind it remains incomplete and is discussed in detail below.

NINJ1 is ubiquitously expressed across mouse tissues; however, protein levels across different cell types within each tissue have not been determined [13]. Several murine cell lines and primary cells, including both immune and non-immune cells, express endogenous NINJ1, and many *in vitro* studies have delineated the role of NINJ1 using murine cells, including bone marrow-derived macrophages (BMDMs), mouse embryonic fibroblasts, and neutrophils. Indeed, myeloid-derived cells express some of the highest mRNA levels of *NINJ1* in both mice and humans. It was recently demonstrated that NINJ1 and its homolog NINJ2 are important for WNT signaling-mediated amplification of hematopoietic stem cells in both zebrafish and humans [14]. Intriguingly, this is independent of NINJ1’s ability to induce PMR. Thus, NINJ1 not only plays a functional role in terminally differentiated immune cells but is also important for their development.

In humans, while RNA sequencing data indicates that *NINJ1* expression is present across most tissues in the body, the level of endogenous protein expression is unknown. Experimental work has demonstrated NINJ1 expression in human monocyte-derived macrophages, several liver cell types, and kidneys by histology [15–17]. In contrast with primary cells, most immortalized human cell lines do not express high levels of (or any) endogenous NINJ1 [13]. Why this differs from mouse cell lines is unclear. Nevertheless, immortalized human cells are known to release large cytoplasmic molecules including lactate dehydrogenase upon lytic cell death (see, for example, [18–20]), raising the question of what molecular mechanism allows this release in human cell lines expressing little to no NINJ1. Overall, limited evidence exists from human systems mechanistically implicating endogenous NINJ1 in PMR, although several recent studies have demonstrated NINJ1 oligomerization from samples taken from ARDS (acute respiratory distress syndrome) and hepatic ischemia-reperfusion injury patients [17,21].

## Mechanism of NINJ1 activation

Since the discovery of NINJ1 as the executioner of PMR, major questions about its biology have included what is the molecular mechanism triggering NINJ1 oligomerization and how NINJ1 oligomers rupture the plasma membrane?

Given that NINJ1 oligomerizes during a diverse range of cell death modalities [3,4], which all require different proteins for their induction and execution, it is assumed that a common event that occurs during all cell death modalities must trigger NINJ1 oligomerization. There are a wide range of events that are common to all forms of lytic cell death, including: Ca^2+^ ion fluxes, lipid scrambling, and osmotic cell swelling [22–24]. All these events have been speculated to trigger NINJ1 oligomerization.

Ca^2+^ influx occurs during many forms of cell death, including apoptosis, pyroptosis, and ferroptosis. One hypothesis is that Ca^2+^ influx triggers NINJ1 oligomerization through the induction of plasma membrane lipid scrambling [15,25]. Indeed, phosphatidylserine (PS) is exposed on the extracellular leaflet of the plasma membrane during all forms of cell death as a result of the activation of lipid scramblases by proteolytic cleavage or increased intracellular Ca^2+^ concentrations [26,27]. One preprint has shown that Ca^2+^ ionophores, such as ionomycin, induce PS exposure and NINJ1 oligomerization in mouse macrophages. Genetic blockade of PS exposure prevented NINJ1 oligomerization and PMR, as did preventing Ca^2+^ influx during pyroptosis. However, ionomycin-induced Ca^2+^ influx can induce necrosis, thereby directly triggering NINJ1, making it essential to control for metabolic cell death in these experiments. A second study has also implicated PS in NINJ1 activation, showing that inhibition of PS exposure during necrosis through the genetic disruption of the lipid scramblase XKR8 decreases NINJ1-mediated PMR [25]. This hypothesis is particularly interesting, as NINJ1 can interact with lipids, especially as a filament, and thus could be poised to detect altered lipid distributions [11]. On the other hand, PS exposure alone might not be sufficient to activate NINJ1, as work by the Nagata lab has shown that forced PS exposure, caused by the expression of a constitutively active TMEM16F, does not result in cell death, although whether NINJ1 is expressed in these cell lines has not been tested [28].

Cell swelling was initially proposed as an activation mechanism for NINJ1 [4]; however, our findings, available as a preprint, suggest that swelling is dispensable for NINJ1 oligomerization and is rather needed for the opening of NINJ1 lesions downstream of oligomer formation [29].

Another consideration has been how the membrane facilitates the molecular rearrangement of NINJ1 that occurs during oligomerization. Molecular dynamics simulations initially suggested that the separation of NINJ1 homodimers would be critical to allow kinking of TM1 for NINJ1 oligomerization [10]. However, a preprint from the Broz lab has proposed that NINJ1 oligomers form through the lateral association of inactive dimers and that dimer separation occurs after oligomer formation [29]. It remains unclear how any of the above-mentioned activation signals would increase lateral association of dimers or bring about dimer separation to form a filament. One hypothesis is that an increase in membrane fluidity during cell death could lead to increased mobility of the dimers, increasing the probability of dimers associating laterally. Alternatively, an accumulation of NINJ1 in the plasma membrane, either through increased expression or retention in the plasma membrane, could push the equilibrium toward higher-order NINJ1 oligomers. In agreement with this, NINJ1 expression is positively correlated with susceptibility to PMR, and several reports correlate negative disease outcomes with increased NINJ1 expression [17,21,30,31]. One recent study observed that cells with higher levels of NINJ1 exhibited increased levels of PMR upon exposure to shearing forces from mechanical strain. This shearing force-dependent PMR was reduced when the K45Q oligomerization-deficient mutant of NINJ1 was expressed, implying that it is the oligomeric form of NINJ1 that weakens the membrane. Furthermore, a recent study identified TRIM72 as an E3 ubiquitin ligase regulating NINJ1 levels through proteasomal degradation. Cells deficient for TRIM72 had increased amounts of NINJ1 and were more susceptible to PMR [16]. Thus, while multiple hypotheses have been put forward to explain the molecular trigger leading to oligomeric NINJ1, definitive experimental evidence has yet to clarify the first step in NINJ1 activation.

Regarding how oligomerization leads to PMR, there have been three models proposed. The initial paper describing the NINJ1 filament structure proposed two possible models for lesion formation [9]. First, a single filament of NINJ1 could form a pore-like lesion through repulsion of lipids, as is observed for other pore-forming proteins like GSDMD [32]. Alternatively, it was proposed that NINJ1 filaments may associate through their hydrophilic surface to form a double filament and that the separation of this hydrophilic interface would unzip the filament to form a lesion. Subsequent studies found that oligomeric NINJ1 is released into the supernatant by dying cells that overexpress NINJ1 and that these oligomers are associated with small lipid discs with an average diameter of 35 nm [11,12]. This observation led to the proposition of the ‘cookie cutter’ model, in which NINJ1 filaments wrap around discs of plasma membrane lipids with their hydrophobic face, exposing the hydrophilic face of the filament to the solvent and solubilizing the discs out of the plasma membrane to leave a lesion behind. Interestingly, a preprint utilized dynamic AFM imaging of NINJ1 in supported lipid bilayers and found that NINJ1 filaments form pore-like structures in the membrane with the filament remaining associated with the membrane. As the lesions grew, they fused, leading to the release of membrane fragments surrounded by oligomeric NINJ1, potentially harmonizing the zipper and cookie cutter models [33].

Overall, while various signals for NINJ1 oligomerization and mechanisms of NINJ1-induced PMR have been proposed, no consensus has emerged.

## NINJ1 in infection and inflammatory disease

The discovery of a single protein mediating cellular rupture captivated the scientific imagination as a means of targeting the negative inflammatory consequences of infection and disease (Table 1). Nevertheless, the full promise of NINJ1 inhibition in alleviating the negative consequences of inflammation across broad disease models is, so far, yet to be borne out. That said, the identification of the role of NINJ1 in lysis is recent, and promising studies are emerging. Efforts to probe the role of NINJ1 in many infectious and inflammatory conditions have been hampered by the hydrocephalus plaguing *Ninj1^−/−^* mouse colonies, leading to small animal numbers. The biological connection between NINJ1 and development of hydrocephalus has yet to be elucidated. Nonetheless, targeting NINJ1 has been shown to have the greatest potential in models of liver disease, and, more recently, an emerging role for NINJ1 in the recruitment of innate immune cells and the generation of an antibody response during tumorigenesis has been observed [34–37].

**Table 1:** A collection of disease contexts where NINJ1 has been shown to play a role, including the model or pathogen, the contribution of NINJ1 to disease, and the cellular or organismal system employed

Disease context	Is NINJ1 presence protective or detrimental?	Model(s) used	Role/effect of NINJ1	References
**Infection**				
*Citrobacter rodentium*	Protective	*in vivo* (mouse)	Improved survival in the presence of NINJ1	[3]
*Yersinia pseudotuberculosis*	Protective	*in vivo* (mouse)	Improved survival in the presence of NINJ1	[38]
*Mycobacterium tuberculosis*	Unknown	Human macrophages	NINJ1 mediates PMR through a Mtb effector ESX-1	[39]
*Candida albicans*	Unknown	Mouse macrophages	NINJ1 allows release of hyphae	[40]
*Salmonella typhimurium*	Protective	Zebrafish embryos	Modulates larval susceptibility by regulating hematopoiesis	[14]
Influenza A virus	Detrimental	*in vivo* (mouse) Mouse macrophages Human alveolar epithelial cells	Improved survival in the absence of NINJ1	[41]
Murine norovirus	Unknown (likely detrimental)	Mouse microglia	NINJ1 increases virion release and secretion of the viral protein NS1	[42,43]
Herpes simplex virus 1	Protective	Mouse macrophages	NINJ1 prevents HSV-1 entry leading to increased cytokine secretion	[13]
**Inflammation**				
Liver disease (Hepatitis & Ischemia-reperfusion injury)	Detrimental	*In vivo* (mouse and rats), GWAS	Removing or blocking NINJ1 improves liver health in various rodent models	[6,16,17,44–46]
Acute kidney injury and chronic kidney disease	Detrimental	*In vivo* (mouse) Human biopsies, Renal Epithelial cells	Improved kidney health upon NINJ1 removal	[16,47,48]
Gout	Detrimental	GWAS study	NINJ1 mutations associated with Gout	[49]
Pulmonary fibrosis	Inconclusive	*In vivo* (mouse) Human lung tissue	NINJ1 expression up-regulated during pulmonary fibrosis	[31]
Acute lung injury and ARDS	Detrimental	*In vivo* (mouse) and *ex vivo* (human)	Neutrophil specific knockout improves survival	[21]
LPS heat shock	Detrimental	Mouse macrophages *In vivo* (mouse)	Improved survival and reduced cell death in the absence of NINJ1	[50]
Sepsis	Detrimental	*In vivo* (mouse)	Uses an inhibitor of NINJ1 oligomerization or NINJ1 heterzygous animals	[51,52,53]
Multiple sclerosis	Detrimental	*In vivo* (mouse) Human brain	Mediates immune cell extravasation	[35,36]
**Cancer**				
Bladder and breast	Detrimental	*In vivo* (mouse)		[34]

During infection, NINJ1 has been shown to play both a beneficial and detrimental role, depending on the infectious context. On the beneficial side, the hypothesis is that NINJ1 activation generates a positive host inflammatory response that is important for resolving infection. Indeed, NINJ1-deficient mice infected with *Citrobacter rodentium* or *Yersinia pseudotuberculosis* exhibited decreased survival, suggesting a beneficial role of NINJ1 for the host in these contexts [3,38]. However, these results have yet to be repeated and extended by others in the field. Notably, it has not been formerly proven if it is the DAMPs released by NINJ1-mediated PMR that drive this protection, for example, by activating defensive programs in bystander cells, or if other mechanisms play a role. Additionally, we have described a novel role for NINJ1 in limiting entry of Herpes Simplex Virus 1 (HSV-1) in mouse macrophages, resulting in a suppressed viral lifecycle and increased cytokine production [13]. This role was independent of NINJ1’s role in cell death, implying that NINJ1 could have pleiotropic roles in the anti-pathogen response.

On the other side, NINJ1 has been shown to be detrimental in models of infection or sterile inflammation mimicking infection. Specifically, genetic knockout of *Ninj1* promoted mouse survival in an influenza A infection model [41], as it did in injection models of Fla-Tox, a purified component of bacterial flagellin known to trigger the NAIP-NLRC4 inflammasome, in *Ninj1* heterozygote animals with reduced NINJ1 expression [52]. Loss of NINJ1 was also protective in a sepsis model of LPS injection followed by heat stress [50]. All these examples would be candidates where targeting NINJ1 should be protective for disease, potentially suppressing an overexuberant immune response that has detrimental consequences for the organism.

In cell culture models, NINJ1 oligomerization has additionally been shown to be triggered by pathogens to facilitate their exit from cells. *Murine norovirus* (MNoV), *Candida albicans*, and *Mycobacterium tuberculosis* (Mtb) were all shown to trigger NINJ1-mediated PMR with beneficial effects for the pathogen. This was demonstrated in the case of MNoV and *C. albicans* and hypothesized for Mtb [39,40,42,43]. Thus, like many other key cell death and innate immune proteins, pathogens have found ways of co-opting NINJ1 for their own benefit, often by triggering NINJ1 activation through damaging the plasma membrane using pathogen-encoded pore-forming proteins.

Outside of the context of pathogens, NINJ1 has most convincingly been implicated in liver disease. The outcomes of models of viral hepatitis and sterile liver damage were improved after genetic ablation of NINJ1 or the targeting of NINJ1 using neutralizing antibodies, which prevent its oligomerization [6]. A recent study has further shown NINJ1 expression in human and mouse hepatocytes and Kupffer cells, with NINJ1 ablation being protective during ischemia-reperfusion injury in mice and rats as measured by circulating liver enzymes and histology [17]. This study also observed increased NINJ1 expression in mice and humans after liver injury, a finding that echoes the initial discovery of NINJ1 as being up-regulated after axonal injury and more recent findings that NINJ1 levels correlate with activation [30]. Finally, NINJ1 expression and oligomerization have been identified in human and mouse neutrophils, and neutrophil-specific knockout of NINJ1 protects mice from pathology and mortality in a model of acute lung injury [21,54].

Since the identification of NINJ1 as the final executioner of PMR in multiple cell death pathways, it has been assumed that the effects of NINJ1 are due to the release of pro-inflammatory cytoplasmic contents. To this end, mass spectrometry analyses have interrogated proteins released in a NINJ1-dependent manner during various types of lytic cell death [3,5]. HMGB1 has long been recognized to be released from dying cells, and it is now appreciated that this occurs in a NINJ1-dependent manner [3]. While treatment of mouse macrophages with purified HMGB1 is not sufficient to elicit an immune response [55], it is likely that it is the complex of HMGB1 with other molecules including DNA or nucleosomes that is pro-inflammatory and can contribute to autoimmune conditions including SLE [56]. Similarly, DNA is known to be released during cell death [57] and has recently been reported to be released in a NINJ1-dependent manner [], although the consequences of this DNA release have not been explored. Thus, to date, the evidence for soluble NINJ1-dependent factors eliciting an immune response by acting as a direct trigger(s) of pattern recognition receptors has not been definitively demonstrated.

While most cytokines are released via the Golgi-secretory system axis, some non-canonically released cytokines depend on alternative mechanisms, including cell death, for release. IL-1β, for example, is released following Caspase-1 processing and exits through Gasdermin D pores [58]. IL-1α is part of the same IL-1 family as IL-1β but has been reported to be released by cells undergoing lytic cell death [59,60]. One recent report has proposed that IL-1α is released in a NINJ1-dependent manner and contributes to an antibody response. In this paper, chemotherapy-killed dead cells were injected into mice as a vaccine. Seven days later, live cancer cells were reinjected into the same animals. WT animals developed tumors that the animals succumbed to, while *Ninj1^−/−^* animals developed smaller tumors and had increased survival. This mechanism was attributed to NINJ1-dependent IL-1α release, which led to neutrophil recruitment, generating a CD8 T-cell response [34]. Another report observed NINJ1-independent release of IL-1α in mouse BMDMs after the induction of pyroptosis, indicating there may be cell type-dependent specificities in its release [3]. IL-1α is not the only cytokine proposed to be released in a NINJ1-dependent manner; IL-33 has also been shown to be released by NINJ1 lesions in pyroptotic and apoptotic mouse immortalized BMDMs [68]. Thus, while dispensable for canonical cytokine release, NINJ1 may contribute to cytokine release in some contexts.

A role for NINJ1 in recruiting and interacting with immune cells is a new theme emerging in the NINJ1 literature. When NINJ1 was exclusively known to be important for adhesion, a role in cell migration was already suggested [35–37]. More recently, the loss of NINJ1 has been shown to result in reduced monocyte and neutrophil infiltration in various models including influenza A virus infection, acute lung injury, and a folic acid injection model of acute kidney disease [16,21,41]. Once immune cells are recruited to the site of injury, NINJ1 may play an additional role in revealing molecules within dying cells. Specifically, NINJ1 was shown to contribute to the recognition of dying cells by dendric cells (DCs) through their F-actin receptor CLEC9A, suggesting that NINJ1 lesions may help DCs sample the interior of dead cells, with consequences for antigen presentation and antibody generation [61].

## Future directions

The discovery of NINJ1-mediated PMR in the early 2020s changed how the field thinks about this process. Moving forward, we identify three significant areas of NINJ1 biology to explore: (1) defining the trigger for NINJ1 oligomerization; (2) investigating the effect of NINJ1-mediated PMR on bystander cells; (3) fully understanding the disease-relevant contexts where therapies targeting NINJ1 could be beneficial.

As previously mentioned, the trigger for NINJ1 oligomerization remains poorly defined, and its identification could provide a novel therapeutic axis to explore. Further experiments probing the role of ion influx and lipid scrambling in the plasma membrane during all forms of cell death where NINJ1 is implicated will be critical for validating the results presented in existing preprints [15,25]. Additionally, given that SIGLEC12 activation to execute PMR during necroptosis requires changes in post-translational modifications as well as cleavage, careful analysis of whether such events are also required in the context of NINJ1 is needed [62].

Many of the DAMPs that are released by NINJ1-mediated PMR have previously been reported to activate bystander cells and generate inflammation [22,63]. However, at present there has been no systematic analysis of the consequences of NINJ1 deficiency on the immunogenicity of cell death nor an investigation of the role of cell-associated NINJ1 compared with soluble proteins released in a NINJ1-dependent manner. It will be interesting to understand what role NINJ1-mediated DAMP release plays in activating both immune and non-immune bystander cells, especially comparing its importance during different forms of cell death including pyroptosis, ferroptosis, and secondary necrosis. As pyroptosis releases highly inflammatory molecules such as IL-1β cytokines through NINJ1-independent mechanisms [3], it is possible that NINJ1-mediated DAMP release may be more important for generating an inflammatory response during forms of cell death where other membrane pores are not formed, such as ferroptosis [5].

The pathogenesis of autoinflammatory and autoimmune conditions is often complex and multifactorial. The release of large DAMPs has been reported to be elevated in many of these conditions, and targeting DAMPs such as HMGB1 with antibodies ameliorates disease in several inflammatory disease models [64]. Moreover, autoimmune conditions such as systemic lupus erythematosus and Sjogren’s syndrome are characterized by antibodies against nuclear components, especially anti-nuclear antibodies, anti-dsDNA, and anti-Sm antibodies [65–67]. Whether NINJ1 plays a role in nuclear antigen release for uptake and presentation by antigen-presenting cells, a critical step in autoantibody generation, is unknown. Of equal importance will be follow-up studies on the promising reports demonstrating that NINJ1 deficiency or inhibition of oligomerization confers protection in models of liver and kidney inflammation to define how this protection is achieved and how broadly applicable the protection is.

## Perspectives

- The discovery of NINJ1 changed how the field thought about the process of PMR; if one protein is responsible for rupturing the membrane, the resulting inflammation could potentially be prevented.
- Structural understanding of NINJ1 has advanced significantly, resulting in a clear picture of the structural rearrangement that occurs during oligomerization. After cell death, NINJ1 has been assumed to be important for the release of soluble factors, although it also seems to be important for the recruitment of immune cells. In infection models, NINJ1 is important for the release of some pathogens. On the host side, targeting NINJ1 in liver disease in mice is so far the most promising.
- Key future directions include (1) defining the trigger for NINJ1 oligomerization; (2) investigating the effect of NINJ1-mediated PMR on bystander cells; and (3) fully understanding the disease-relevant contexts where therapies targeting NINJ1 could be beneficial.

## Open Access

Open access for this article is provided via our Subscribe to Open programme, made possible through the support of our subscribing institutions. Learn more here: https://portlandpress.com/pages/open_access_options_and_prices#conditional.

## Abbreviations

ARDS: acute respiratory distress syndrome
BMDMs: bone marrow-derived macrophages
DAMPs: damage-associated molecular patterns
DCs: dendric cells
HMGB1: high mobility group box 1 protein
HSV-1: Herpes Simplex Virus 1
MNoV: Murine norovirus
Mtb: Mycobacterium tuberculosis
NINJ1: Ninjurin-1
PMR: plasma membrane rupture
PS: phosphatidylserine
TM1: first transmembrane helix
